# Do faecal test-based colorectal cancer screening pilots provide data that are reflected in subsequent programmes? Evidence from interval cancer proportions

**DOI:** 10.1177/00045632221119714

**Published:** 2022-08-27

**Authors:** Gavin RC Clark, Robert JC Steele, Callum G Fraser

**Affiliations:** 1 9571 Public Health Scotland, Edinburgh, UK; 2 Centre for Research into Cancer Prevention and Screening, 85326 University of Dundee, Dundee, UK

**Keywords:** Colorectal cancer screening, faecal immunochemical test, faecal occult blood test, interval cancers

## Abstract

**Objective:**

Guidelines on colorectal cancer (CRC) screening with guaiac faecal occult blood tests (gFOBTs) and faecal immunochemical tests (FITs) include the need for a pilot before a programme is introduced. Interval cancers (ICs), cancers arising after a negative screening test result but before the next scheduled invite, are important indicators of programme quality. Our aim was to compare IC in the gFOBT-based Scottish Bowel Screening Programme (SBoSP), a FIT-based pilot, and the FIT-based SBoSP, to assess if the pilot provided data that was reflected in the subsequent programme.

**Design:**

The IC proportions (ICPs) data ([IC/(IC + screen detected CRC)] x 100) from the penultimate year of the gFOBT-based SBoSP, the 6-month pilot and the first year of the FIT-based SBoSP were compared. To ensure appropriate comparison, these data were only from the two pilot NHS Boards.

**Results:**

For all participants, and females and males, the ICPs were very similar in the gFOBT-based SBoSP and the pilot. The faecal haemoglobin concentration (f-Hb) threshold for the pilot was set at ≥80 μg Hb/g faeces. However, in marked contrast, in the FIT-based SBoSP, at the same threshold, the ICPs were lower. In all three groups, the ICPs were higher in females than in males.

**Conclusions:**

Data on variables in pilots, including ICP, can be informative, but only if variables such as FIT system are held consistent between pilot and programme. Lowering the f-Hb threshold for females to give the same ICP as males might be a strategy to minimise sex inequality.

## Introduction

European guidelines on testing for the presence of blood in faeces in colorectal cancer (CRC) screening programmes, including guaiac faecal occult blood tests (gFOBTs) and the newer faecal immunochemical tests (FITs), include statements on the need for a pilot to be performed before a screening programme is initiated.^1^ Interval cancers (ICs) are CRC that are detected after a negative screening test result but before the next invitation for screening and are important quality indicators. To our knowledge, Scotland^2^ and the Netherlands^3^ are the only two countries that have reported in detail on the consequences of undertaking a pilot and then identifying whether the data from the pilot were reflected in those from the subsequent programme. In both countries, the data generated in the pilot did inform usefully on certain aspects of the programme, particularly the uptake and logistics, but did not fully reflect the outcomes, including test result positivity, the resulting colonoscopy demand and the clinical outcomes. The aims of this study were (a) to compare IC proportions (ICPs) in all participants, and in females and males, in the gFOBT-based Scottish Bowel Screening Programme (SBoSP), a FIT-based pilot, and the FIT-based SBoSP, to (a) assess if the pilot data were reflected in the programme and (b) to make recommendations on possible future strategies.

## Methods

The SBoSP was initiated in a United Kingdom demonstration pilot running from 2000 to 2002 using biennial gFOBT in the 50–69 year age range. The results of this pilot, along with those from England, were used to inform a UK National Screening Committee decision to recommend national CRC screening programmes across the United Kingdom. After a further two pilot screening rounds, roll-out across the whole of Scotland, still using initial gFOBT, but for a 50–74 age range, started in July 2007 and was complete by December 2009. In 2010, a 6-month pilot evaluation of FIT as the initial investigation was performed in two of the 14 NHS Boards responsible for regional health care in Scotland using a quantitative FIT (OC-Sensor Diana, Eiken Chemical Co., Ltd, Tokyo, Japan) at a faecal haemoglobin concentration (f-Hb) threshold of ≥80 μg Hb/g faeces. In November 2017, FIT was introduced throughout the SBoSP at the same f-Hb threshold using the HM-JACK arc system (Minaris Medical Co., Ltd, Tokyo, Japan). The ICP data from the penultimate year of the gFOBT-based SBoSP, the 6-month FIT-based pilot and the first year of the FIT-based SBoSP were calculated and compared. To ensure appropriate comparison, the data were only from the two NHS Boards that participated in the pilot, NHS Tayside and NHS Ayrshire & Arran.^2^

## Results

Table 1 shows the number of IC, the number of screen-detected cancers (SCDs) and the ICP, as percentages with 95% confidence intervals (CIs) and *p*-values, in all participants and in females and males in the gFOBT-based SBoSP, the FIT pilot and the FIT-based SBoSP. Interval cancer proportions as percentages were calculated as (interval cancers/[interval cancers + screen detected cancers]) x 100: that is: ([IC/(IC + SCD)]) x 100): *p*-values comparing the ICP in the gFOBT- and FIT-based SBoSP to the FIT pilot were calculated using the chi-squared test. The ICP percentages are also shown in Supplemental Figure 1.

**Table 1.** Comparison of interval cancer (IC) numbers (n), screen detected cancer (SDC) numbers (n) and interval cancer proportions (ICPs) (%) with 95% confidence intervals (CIs) and *p*-values* in all participants, females and males in two NHS Boards in guaiac faecal occult blood test (gFOBT) in the Scottish Bowel Screening Programme (SBoSP), the faecal immunochemical test (FIT) pilot and FIT in the SBoSP.

	gFOBT in SBoSP	FIT pilot	FIT in SBoSP
IC: n: All	79	31	58
IC: n: Females	38	16	30
IC: n: Males	41	15	28
SDC: n: All	77	30	84
SDC: n: Females	31	14	35
SDC: n: Males	46	16	49
ICP: %: All (95% CI, *p*-values)	50.6 (42.9–58.4, *p* = 0.90)	50.8 (38.6–62.9)	40.8 (33.1–49.1, *p* = 0.25)
ICP: %: Females (95% CI, *p*-values)	55.1 (43.4–66.2, *p* = 0.95)	53.3 (36.1–69.8)	46.2 (34.6–58.1, *p* = 0.67)
ICP: %: Males (95% CI, *p*-values)	47.1 (37.0–57.5, *p* = 0.93)	48.4 (32.0–65.2)	36.4 (26.5–47.5, *p* = 0.17)

*Comparing ICP (%) in the gFOBT-based SBoSP and in the FIT-based SBoSP with the ICP (%) in the FIT pilot.

## Discussion

It is striking that, for all participants, and females and males, in the gFOBT-based SBoSP and the pilot, the ICPs were numerically very similar and not different statistically. This was planned (and expected), since the f-Hb threshold for the pilot was set at ≥80 μg Hb/g faeces to give the same positivity as the gFOBT-based SBoSP with which the colonoscopy capacity could cope at that time. However, in marked contrast, in the FIT-based SBoSP, although not reaching statistical significance, the ICPs are numerically much lower, as are the *p*-values. There are a number of potential reasons for this, including the unlikely possibilities that the three groups have different demographic characteristics, particularly since the introduction of FIT yielded a higher uptake as compared to gFOBT,^2^ but the most plausible is that the f-Hb distributions in the pilot^4^ and FIT-based SBoSP^5^ differ, the latter being much higher, leading to a higher test positivity and increased numbers of colonoscopies being performed^2^ and, in consequence, lower ICP. This is undoubtedly due to the fact that the FIT analytical systems in the pilot (OC-Sensor) and programme (HM-JACKarc) differed.

Similar findings were experienced in the Netherlands^3^ and a few months’ monitoring of the programme showed that uptake and positivity were higher and the positive predictive value (PPV) was lower than predicted based on extensive pilot studies. To align with colonoscopy capacity, the f-Hb threshold used to initiate further investigation was increased from 15 to 47 μg Hb/g faeces. This decreased the positivity and increased the PPV. The authors concluded that close monitoring of the implementation had allowed optimisation through changing the f-Hb threshold.^3^ In addition to the older age range invited in the programme as compared to the pilot, the FIT analytical system differed between pilot (OC-Sensor) and programme (FOB-Gold, Sentinel Diagnostics, Milan, Italy). In contrast, in the SBoSP, the f-Hb threshold was not changed from the pilot to the programme, in spite of the higher positivity and, consequently, the increased colonoscopy requirement.^2^

In all three groups in our study, the ICP was higher in females than in males and again, this is undoubtedly due to f-Hb being lower in females than in males,^4,5^ so that a smaller percentage of female participants have a screening test result higher than the f-Hb threshold applied, so fewer are referred for colonoscopy, leading to a higher ICP.

In conclusion, pilots can be informative, but only if variables are held consistent between pilot and programme, particularly the FIT systems, since these do give different results.^6^ As we have previously documented, the application of detailed f-Hb distributions is useful and encouraged.^4,5^ Further, in order to reduce the sex inequality between females, who are currently disadvantaged by, *inter alia*, having a higher ICP as compared to males, it may be that lowering the f-Hb threshold for females to give the same ICP as males would be a simple strategy to initiate, although further colonoscopy resources would be required.

## Supplemental Material

Supplemental Material - Do faecal test-based colorectal cancer screening pilots provide data that are reflected in subsequent programmes? Evidence from interval cancer proportions Click here for additional data file. Supplemental Material for Do faecal test-based colorectal cancer screening pilots provide data that are reflected in subsequent programmes? Evidence from interval cancer proportions by Gavin RC Clark, Robert JC Steele and Callum G Fraser in Annals of Clinical Biochemistry

## References

[1] Halloran SP Launoy G Zappa M. International Agency for Research on CancerEuropean guidelines for quality assurance in colorectal cancer screening and diagnosis. First Edition–Faecal occult blood testing. Endoscopy 2012; 44(Suppl 3): SE65–SE87. DOI: 10.1055/s-0032-1309791. 23012123

[2] Clark G Strachan JA Carey FA, et al. Transition to quantitative faecal immunochemical testing from guaiac faecal occult blood testing in a fully rolled-out population-based national bowel screening programme. Gut 2021; 70(1): 106–113. DOI: 10.1136/gutjnl-2019-320297. 32234803

[3] Toes-Zoutendijk E van Leerdam ME Dekker E, et al. Real-time monitoring of results during first year of Dutch colorectal cancer screening program and optimization by altering fecal immunochemical test cut-off levels. Gastroenterology 2017; 152(4): 767–775.e2. DOI: 10.1053/j.gastro.2016.11.022. 27890769

[4] McDonald PJ Strachan JA Digby J, et al. Faecal haemoglobin concentrations by gender and age: implications for population-based screening for colorectal cancer. Clin Chem Lab Med 2011; 50(5): 935–940. DOI: 10.1515/CCLM.2011.815. 22149740

[5] Clark GRC Strachan JA McPherson A, et al. Faecal haemoglobin distributions by sex, age, deprivation and geographical region: consequences for colorectal cancer screening strategies. Clin Chem Lab Med 2020; 58(12): 2073–2080. DOI: 10.1515/cclm-2020-0268. 32324157

[6] Benton SC Piggott C Zahoor Z, et al. A comparison of the faecal haemoglobin concentrations and diagnostic accuracy in patients suspected with colorectal cancer and serious bowel disease as reported on four different faecal immunochemical test systems. Clin Chem Lab Med 2022; 60(8): 1278–1286. DOI: 10.1515/cclm-2021-1248. 35637625

