# Supplementary material for: Do faecal test-based colorectal cancer screening pilots provide data that are reflected in subsequent programmes? Evidence from interval cancer proportions
Source: Ann Clin Biochem. 2022 Aug 27;59(6):450–2. doi: 10.1177/00045632221119714 (PMC9634330; doi:10.1177/00045632221119714)

Supplementary Figure 1. Comparison of interval cancer proportions (ICP) (%) in all participants, women and men in two NHS Boards in guaiac faecal occult blood tests (gFOBT) in the Scottish Bowel Screening Programme (SBoSP), the faecal immunochemical test (FIT) pilot, and FIT in the SBoSP.

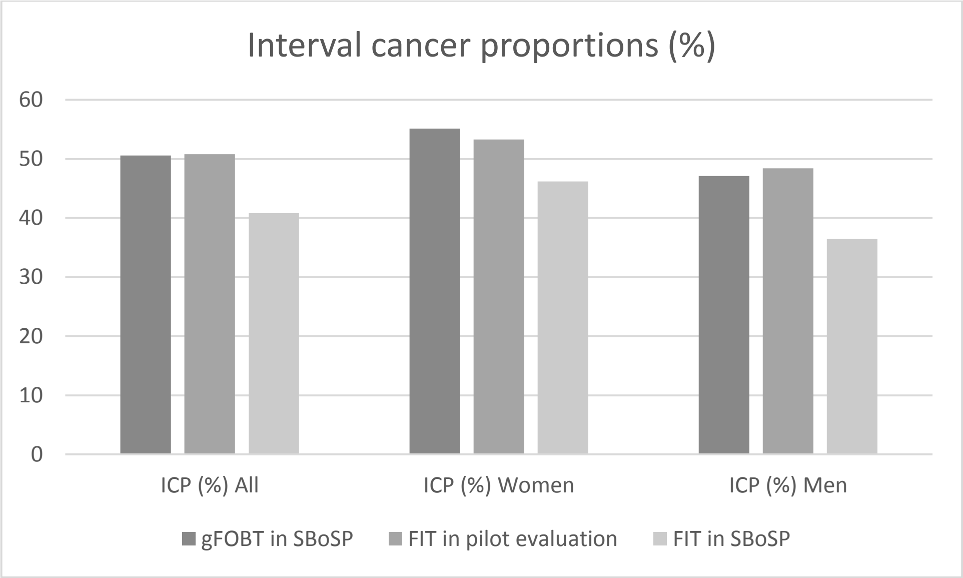

Supplement: Supplemental Material - Do faecal test-based colorectal cancer screening pilots provide data that are reflected in subsequent programmes? Evidence from interval cancer proportions [file sj-pdf-1-acb-10.1177_00045632221119714.pdf]
